# Saturation transfer difference NMR on the integral trimeric membrane transport protein GltPh determines cooperative substrate binding

**DOI:** 10.1038/s41598-020-73443-z

**Published:** 2020-10-05

**Authors:** Jenny L. Hall, Azmat Sohail, Eurico J. Cabrita, Colin Macdonald, Thomas Stockner, Harald H. Sitte, Jesus Angulo, Fraser MacMillan

**Affiliations:** 1 Henry Wellcome Unit for Biological EPR, School of Chemistry, University of East Anglia, Norwich Research Park, Norwich, NR4 7TJ UK; 2 Institute of Pharmacology, Medical University of Vienna, Währingerstrasse 13A, 1090 Vienna, Austria; 3 UCIBIO, Chemistry Department, Faculty of Sciences and Technology, NOVA University of Lisbon, 2829-516 Caparica, Portugal; 4 School of Pharmacy, University of East Anglia, Norwich Research Park, Norwich, NR4 7TJ UK

**Keywords:** Biophysics, Structure determination, NMR spectroscopy, Solution-state NMR, Biophysical chemistry, Mechanism of action, Transporters

## Abstract

Saturation-transfer difference (STD) NMR spectroscopy is a fast and versatile method which can be applied for drug-screening purposes, allowing the determination of essential ligand binding affinities (*K*_D_). Although widely employed to study soluble proteins, its use remains negligible for membrane proteins. Here the use of STD NMR for *K*_D_ determination is demonstrated for two competing substrates with very different binding affinities (low nanomolar to millimolar) for an integral membrane transport protein in both detergent-solubilised micelles and reconstituted proteoliposomes. GltPh, a homotrimeric aspartate transporter from *Pyrococcus horikoshii*, is an archaeal homolog of mammalian membrane transport proteins—known as excitatory amino acid transporters (EAATs). They are found within the central nervous system and are responsible for fast uptake of the neurotransmitter glutamate, essential for neuronal function. Differences in both *K*_D_’s and cooperativity are observed between detergent micelles and proteoliposomes, the physiological implications of which are discussed.

## Introduction

L-glutamate is the primary excitatory neurotransmitter in the mammalian central nervous system. Excitatory amino acid transporters (EAATs) are membrane proteins that play an important role in regulation of the glutamate concentration within the synaptic cleft of glutamatergic neurons, thereby terminating synaptic transmission. Efficient uptake prevents a build-up of high extracellular glutamate levels leading to excitotoxicity (one of the hallmark features of Amyotrophic lateral sclerosis (ALS)), and also allows glutamate to be recycled^1^. Substrate transport by EAATs is energised by the co-transport of up to three Na^+^ ions and one proton. The counter-transport of one K^+^ ion completes the putative transport cycle^2,3^. In addition, EAATs exhibit uncoupled chloride-conductance, making them anion-selective ion channels^4^. Various neurological diseases are linked to their dysfunction in glutamatergic synapses, including ALS, stroke, epilepsy and Huntington’s disease^1^.

**Figure 1:**
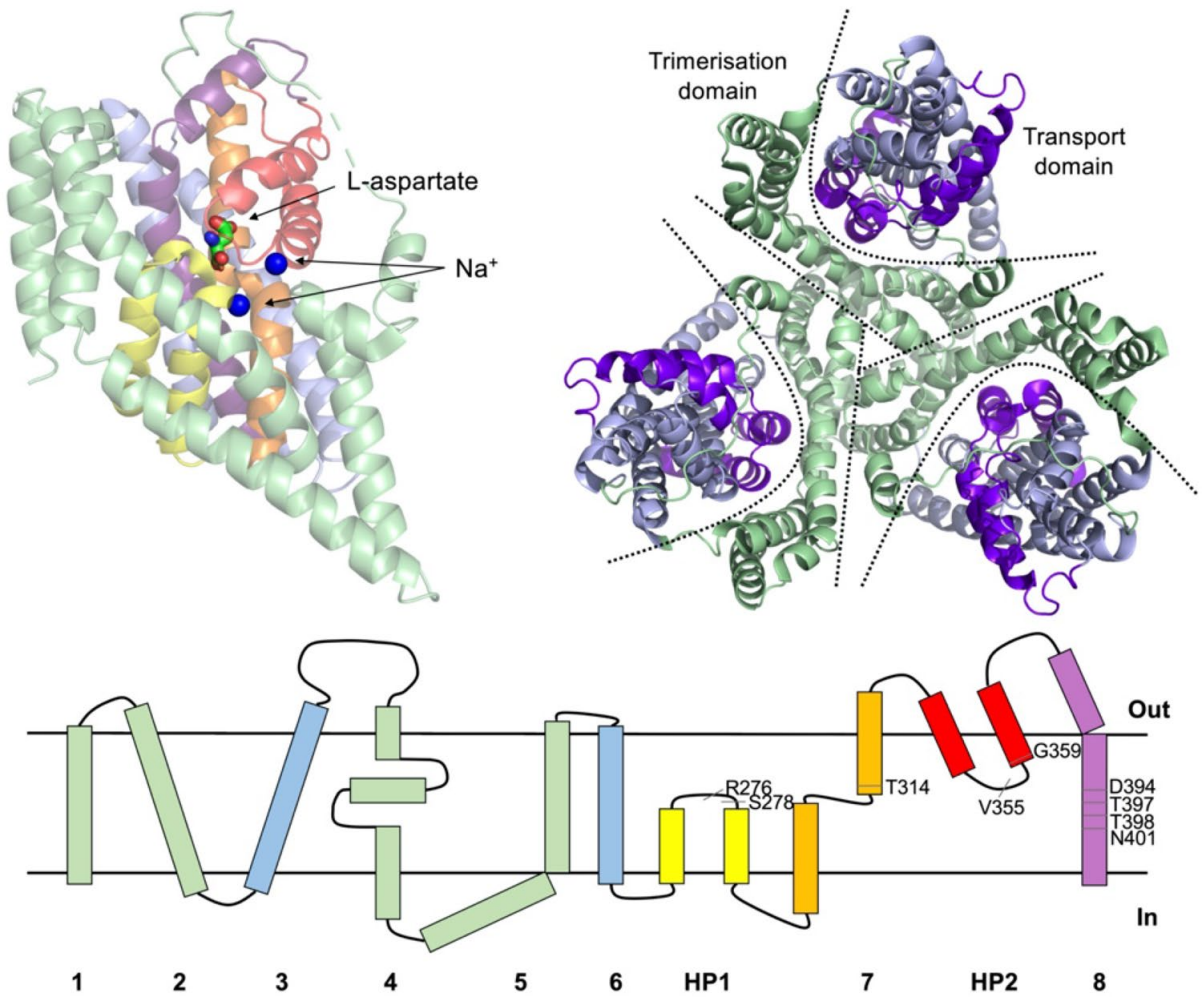
(Top) left: Cartoon representation of a single GltPh protomer with bound aspartate (sticks) and two Na^+^ ions (blue spheres). HP1, TM7, HP2 and TM8 containing ligand binding residues are shown in yellow, orange, red and violet respectively. (Top) right: extracellular view of GltPh with trimerisation domains shown in green and transport domains in blue. Individual protomers are separated by straight lines, whilst transport domains are separated from trimerisation domains by curved lines. (Bottom) a cartoon representation of the GltPh fold highlighting the residues involved in substrate binding. The protein images were prepared using PyMOL (The PyMOL Molecular Graphics System; https://www.pymol.org).

GltPh, a bacterial homolog of mammalian glutamate transporters from *Pyrococcus horikoshii* was the first member of the Solute Carrier 1 (SLC1) family of membrane transporters to have been crystallized while very recently a first crystal structure of human EAAT1 was determined facilitating extrapolation of results obtained for GltPh to its human counterparts^5–8^. GltPh is often used to investigate structure–function relationships of the SLC1 transporter family via spectroscopic techniques including electron paramagnetic resonance (EPR) spectroscopy and single molecule fluorescence resonance energy transfer (smFRET)^9^. It shares approximately 35% amino acid sequence identity with human EAATs; and importantly, many of the amino acids involved in ion and substrate binding are conserved, making it an excellent model system^10^. The outward-occluded state, captured as the first crystal structure of GltPh, revealed a homotrimer assembly with a bowl-shaped extracellular basin whose surface is hydrophobic and reaches halfway down the trimer’s height (Fig. 1)^6–8^.

Each protomer consists of a trimerisation domain involved in stabilising subunit interactions, and a transport domain, containing the substrate and sodium ion binding sites. Further crystal conformations have been solved in the outward-open^10^, inward-occluded^11,12^, as well as intermediate^13^ conformations, revealing large-scale translational movement upon substrate binding and transport. The substrate binding site (Fig. 1) is formed by the tips of the two re-entrant loops (HP1 and HP2) and unwound regions of transmembrane (TM) helices 7 and 8^10^.

Whilst membrane proteins such as GltPh account for one-third of all proteins encoded by the genomes of most organisms^14^, they continue to prove challenging for structural and mechanistic studies. This difficulty extends to include medium to high throughput determination of substrate and inhibitor binding affinities, essential for drug target screening. Until now methods such as fluorescence-based assays^10,15,16^, isothermal titration calorimetry^12,15,17^ and uptake experiments^18^ have been utilised in the determination of substrate *K*_M_ and *K*_D_ values in both detergent-solubilised and membrane-reconstituted protein environments. Such methods have yielded results that vary considerably with Na^+^ concentration, clearly highlighting GltPh’s sodium dependency for substrate binding. *K*_D_ values for L-aspartate vary from 2–380 nM depending on Na^+^ concentration (200 mM and 10 mM respectively), while for the much weaker binding ligand, L-glutamate, they vary between 120–250 μM in 200 mM Na^+^.

Here we present a novel approach for the determination of *K*_D_ values for both L-aspartate and L-glutamate using saturation transfer difference (STD) NMR spectroscopy^19^. STD NMR is a sensitive method for ligands of medium to weak affinity, relying on the exchange between bound and free states of the ligand (*K*_D_ values typically ranging from high nM to low mM). During the STD NMR experiment, resonances of the receptor molecule are selectively saturated, and this saturation rapidly spreads via spin diffusion throughout the protein. A ligand which binds to this protein (for between 0.1 to 100 ms) will receive saturation to some of or all its protons, with those in closest proximity to the protein receiving the most^20^. When the ligand dissociates, this saturation is transferred to the bulk solution, where a reduction in ligand NMR signal intensity is observed. The difference between a spectrum without saturation (reference spectrum), and the saturated experiment gives rise to the STD spectrum, in which only signals of ligand molecules that interacted with the protein are observed^21^. The work presented here significantly extends the application of STD NMR to membrane proteins and demonstrates its use as an alternative, fast method for determination not only of *K*_D_ values for weakly binding ligands but also for very tightly binding ligands, not previously thought accessible through STD NMR. In addition to demonstrating that these two ligands share the same binding site, negative cooperativity for the trimeric GltPh in a near native proteoliposome-reconstituted environment is observed.

Current STD NMR literature reporting the direct observation of substrate binding and interaction with membrane proteins is limited to a very small number of systems. Previous examples have included the basic observation of a protein–ligand interaction in whole cells^22–25^, cell membranes^26^ and in proteins reconstituted into proteoliposomes^27^ and nanodiscs^28^. Only very recently have the first examples of STD NMR-based analysis of ligand binding to detergent-solubilised proteins been reported^29–32^. There is only one previous report of using STD NMR for a ligand *K*_D_ determination to a membrane protein which employed an as-yet unspecified and unpublished fitting routine, whereby an STD NMR effect was measured as a function of ligand concentration for a receptor embedded into a proteoliposome^27^.

The three protomers within the trimeric GltPh each have one substrate binding site and they have been previously proposed to function independently^33–36^. Using STD NMR we have directly determined the affinity of the weak binding ligand L-glutamate for GltPh in both detergent micelles and proteoliposomes, whilst binding of the high affinity ligand L-aspartate was identified using a competition-based STD NMR experiment.

## Results and discussion

**Figure 2:**
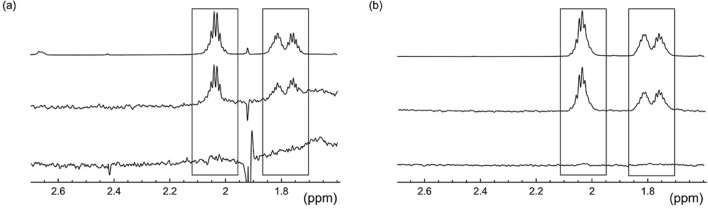
Selected spectral region of the reference ^1^H NMR (top) and corresponding STD NMR spectrum of GltPh incubated with 0.5 mM L-glutamate in the presence (middle) and absence (bottom) of 100 mM Na^+^ ions; (**a**) detergent solubilised GltPh (~ 20 μM) and (**b**) GltPh (~ 20 μM) reconstituted into proteoliposomes (800 MHz, 5 °C, 2 s saturation time). Full spectra can be found in the Supporting Information.

^1^H-NMR spectra of GltPh were measured in the presence of L-glutamate both in detergent (Fig. 2a top) and reconstituted into proteoliposomes (Fig. 2b top). Spectra of detergent-solubilised samples are dominated by strong signals from detergent protons (see Supporting Information). The γ-protons of L-glutamate have resonances occurring at 2.05 ppm, whereas β-protons appear at 1.8 ppm, both in a spectral region devoid of interferences from these strong detergent signals (Fig. 2a). STD NMR signals from γ- and β-protons of L-glutamate are observable in the presence (middle) but not in the absence (bottom) of Na^+^. We observe a similar Na^+^ dependence for GltPh in detergent micelles and in reconstituted proteoliposomes, which is in accordance with previously published data showing that binding and transport of substrate only occurs in the presence of Na^+^. The STD NMR spectra of detergent-solubilised GltPh also reveal signals arising from detergent molecules as a result of two mechanisms: on–off exchange with the protein/detergent macromolecular assembly in solution, and direct saturation through the on-resonance radio frequency. A lack of ligand STD signal in the absence of Na^+^, however, clearly provides evidence that the ligand only receives its saturation when bound to the protein. Any build-up of saturation is limited by relaxation (R_1_ = 1/*T*_1_) of saturated ligand protons in the free state; protons with a longer *T*_1_ accumulate more saturation in the bound state and decay more slowly in solution and hence their relative STD intensity is higher. Since proton relaxation rates are sensitive to molecular tumbling in solution which can be affected by the presence of detergent micelles or proteoliposomes, we determined proton *T*_1_ values for L-glutamate under both conditions (Figure S6). The fitted data shown in Figure S6 indicate that the *T*_1_ values remain the same under both conditions (Table S1).

The method for *K*_D_ determination requires monitoring of ligand STD NMR signals as a function of the ligand concentration, via ligand titration to a large excess. A GltPh STD NMR spectrum in the presence of Na^+^ is recorded initially without L-glutamate. Subsequently, the ligand is titrated incrementally, and further STD NMR spectra are recorded. The experimentally observed STD effect is analysed through conversion to the STD amplification factor (STD-AF) using Eq. (1)^20^

1 $$STD-AF= \frac{{I}_{0}-{I}_{SAT}}{{I}_{0}}\times \frac{{\left[L\right]}_{Total}}{[{P]}_{Total}}$$

where (*I*_0_−*I*_SAT_) represents the ligand signal intensity in the STD NMR spectrum, *I*_0_ is the ligand peak intensity in an off-resonance ^1^H-NMR spectrum (reference) and ([*L*]_Total_/[*P*]_Total_) is the ligand excess relative to a fixed and constant protein concentration.

**Figure 3:**
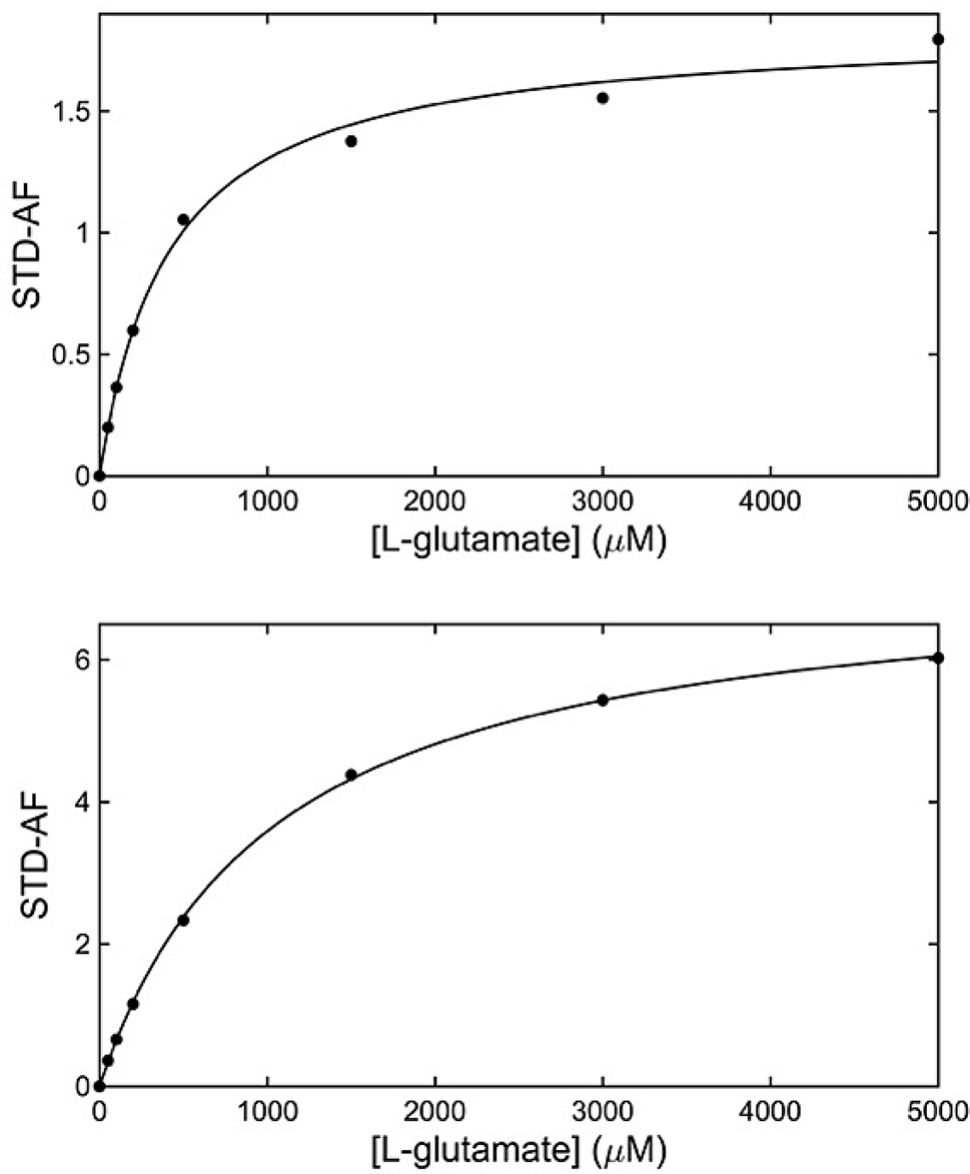
STD amplification factor as a function of L-glutamate concentration for GltPh in detergent micelles (top) and proteoliposomes (bottom). The solid line represents the best fit according to Eq. (3).

The STD-AF for each titration point was determined for both the γ-protons at 2.028 ppm and β-protons at 1.809 ppm and plotted against the concentration of L-glutamate. The resulting hyperbolic curve (using the γ-protons) has an expected decrease in slope at higher ligand concentrations, eventually reaching a plateau caused by saturation of the GltPh binding site (Fig. 3). To utilise this STD-AF for *K*_D_ determination, an equation analogous to the Langmuir isotherm is used (Eq. 2). Here, α_STD_ is the maximum STD-AF and [*L*] is the total concentration of free ligand.

2 $$STD-AF= \frac{{\propto }_{STD}\left[L\right]}{{K}_{D}+\left[L\right]}$$

Determination of the *K*_D_ is simplified when [*L*] = [*L*]_Total_, which is true under the experimental conditions used in STD NMR experiments since [*L*]_Total_ >  > [*P*]_Total_. The STD-AF will increase with increasing [*L*]_Total_ until a maximum value is reached. At this point, [*L*]_Total_ >  > *K*_D_ and the protein binding sites are fully occupied^37^. Strictly speaking in the case of GltPh, since the ligand can bind from both sides of the transporter and STD is more sensitive to the off rates, then our data is reporting on an apparent *K*_D_, instead of a *K*_M_, because the glutamate on/off rates are still an order of magnitude faster than transporter isomerization which is linked to the transport event (i.e. *K*_M_).

α_STD_ and *K*_D_ for L-glutamate are determined by fitting the observed experimental curve using Eq. (2), and the resulting *K*_D_ values for L-glutamate are 414 and 1034 μM for detergent-solubilised and proteoliposome-reconstituted GltPh respectively (Fig. 3) using the γ-protons (very similar results of 458 μM and 984 μM respectively were obtained if the β-protons were used). These values are higher than those previously observed in detergent micelles (between 120–250 μM)^10,16^, however it must be noted that the sodium ion concentration used here is half of that used in those previous studies. It is also known that due to fast protein–ligand rebinding in solution, *K*_D_ values obtained from STD NMR are always greater than, or equal to, the true thermodynamic value^38^. In order to combat such over-estimations, a more involved protocol can be employed in which initial growth rates of each STD amplification factor are determined and used for calculation of binding isotherms for *K*_D_ determination; this application however is not suitable for somewhat unstable membrane proteins^38^. It is therefore expected that the values determined here likely reflect the upper limits of ligand *K*_D_’s.

STD NMR is of limited use for the direct determination of *K*_D_ values of high-affinity ligands (i.e. *K*_D_’s < μM) since tight ligand binding results in low *k*_off_ rates, thus precluding the saturation of the bound ligand being effectively transferred into solution and thus no STD NMR effect is observed. No STD NMR effect from L-aspartate (in the presence of Na^+^) is observed for GltPh which is clearly consistent with its expected much higher binding affinity.

**Figure 4:**
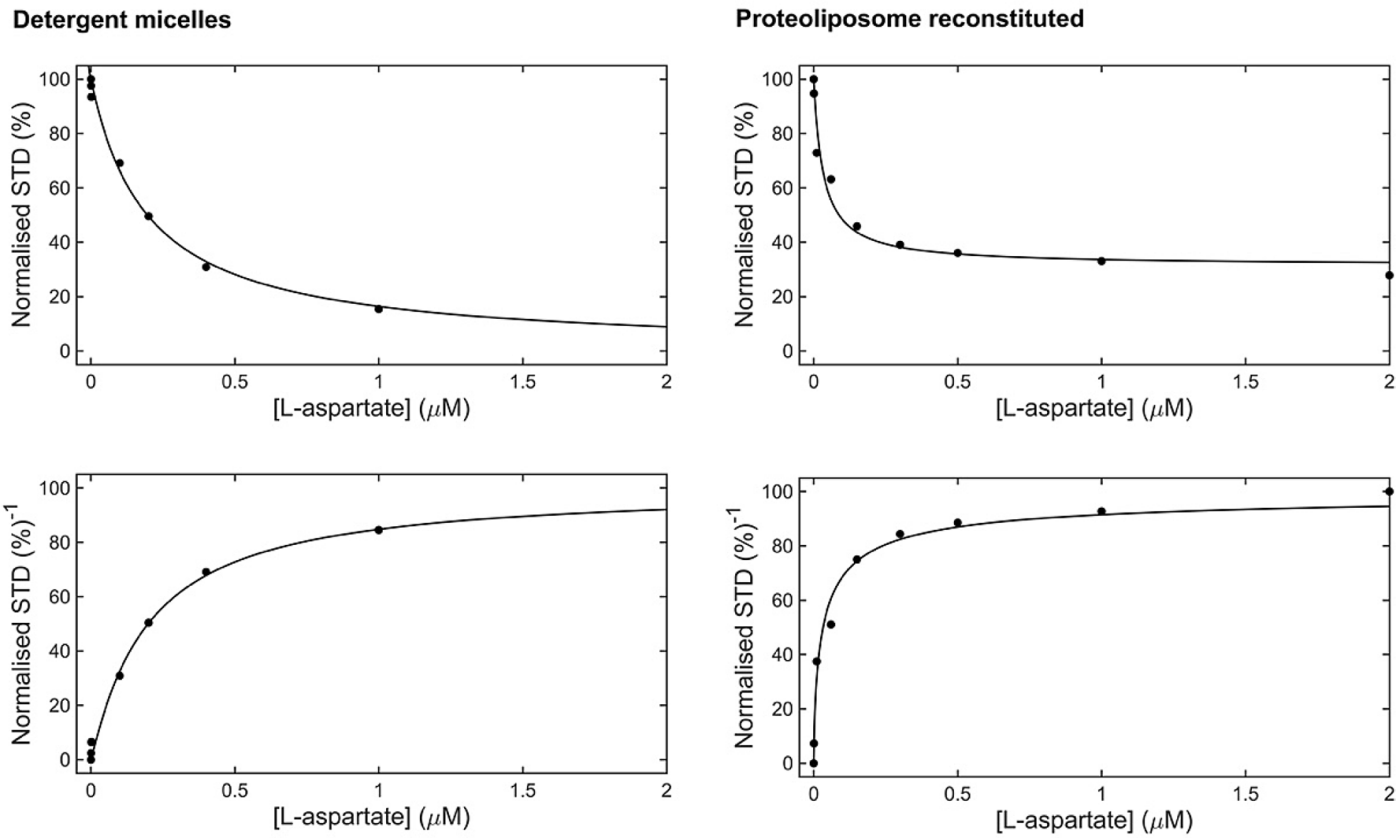
Competition binding of L-aspartate in the presence of excess L-glutamate (1.5 mM) in GltPh. (Top) Normalised STD effect (%) as a function of L-aspartate concentration for GltPh in detergent micelles (left) and reconstituted proteoliposomes (right). The solid line represents the best fit according to Eq. (3). (Bottom) Normalised data fitted to a standard Hill equation (Eq. 4), resulting in Hill coefficients of 1.06 and 0.69 in detergent micelles (left), and reconstituted proteoliposomes (right) respectively.

To overcome such a limitation, a competition experiment may be performed where detection of high-affinity ligand binding is identified via observation of a reduction in the STD NMR signal of a weakly binding so-called ‘reporter’ ligand^39^. Using such a competitive displacement approach we determined an upper limit for the *K*_D_ values of the biologically more relevant, high-affinity GltPh substrate L-aspartate in both detergent-solubilised and proteoliposome-reconstituted environments. In contrast to the initial experiment a titration of L-aspartate is performed on a sample containing a large excess of L-glutamate (1.5 mM) relative to both its previously determined *K*_D_ and the protein concentration. Upon subsequent titration with L-aspartate, a reduction of the STD NMR effect arising from the L-glutamate reporter ligand is observed. The reduction implies the specific displacement of L-glutamate, most probably from the same binding pocket, by the higher affinity ligand L-aspartate. In Fig. 4 the STD effect for L-glutamate, determined by comparison of the reference and STD spectra, and then normalised (%), is recorded against a titration of L-aspartate resulting in a hyperbolic curve which can be fitted directly using Eq. (3)^40,41^. Here, *A* is a proportionality constant, ${K}_{{D}_{glu}}$ the dissociation constant measured for L-glutamate and *L*_*0*_ its fixed concentration, *I*_*0*_ is the total concentration of L-aspartate and ${K}_{{D}_{asp}}$ is its dissociation constant.

3 $$\% bound\propto -A \times \frac{[\frac{{K}_{{D}_{glu}}}{{L}_{0}}][\frac{{I}_{0}}{{K}_{{D}_{asp}}}]}{1+[\frac{{K}_{{D}_{glu}}}{{L}_{0}}][1+(\frac{{I}_{0}}{{K}_{{D}_{asp}}})]}+100$$

By fitting the experimental titration data (Fig. 4) *K*_D_ values for L-aspartate of 42 nM and 13 nM for detergent-solubilised and proteoliposome-reconstituted GltPh were determined respectively. These are comparable to previously estimated values, also in the nM range^10,12,15–18^. A comparison of the *K*_D_ values determined in detergent and proteoliposomes reveals interesting insight into the protein function. The observed values for the weakly binding ligand, L-glutamate, are comparable in magnitude but vary slightly from those observed previously. The concentration of sodium ions used here is lower than used in most previous cases^10,16^, which might explain the slightly higher *K*_D_ values determined here. Indeed, to test this hypothesis a further set of STD NMR measurements were carried out as a function of Na^+^ concentration, using detergent-solubilised WT GltPh in the presence of a fixed, excess concentration of L-glutamate. The STD effect increases with excess Na^+^ concentration (Figure S7) which can be explained by an decrease in *K*_D_ in accordance with previous biochemical data^15^, further highlighting the sensitivity of this particular technique to the system at hand. This serves as an additional control for the Na^+^ dependency of substrate binding.

For the tightly binding L-aspartate ligand there is little difference in the observed *K*_D_ values between protein environments, however upon closer inspection of the titration data in Fig. 4 a distinct difference can be seen. The value of A used in Eq. 3 to fit the experimental data is different for detergent and proteoliposomes. Whereas in detergent the tightly binding ligand fully displaces the L-glutamate (A = 100), in proteoliposomes this value is only 67 suggesting that L-aspartate is not able to fully displace the L-glutamate in GltPh reconstituted into proteoliposomes. To further understand this observation, data from both competition experiments were normalized and subsequently fitted to a standard Hill equation (Fig. 4 bottom) as described by Eq. (4), where *n* is the Hill coefficient.

4 $$y=\frac{1}{\left[{\left(\frac{{K}_{D}}{[L]}\right)}^{n}+1\right]}$$

The Hill coefficient calculated in detergent micelles was 1.063, indicative of typical non-cooperative, independent ligand binding in the trimeric GltPh. In proteoliposomes, however the Hill coefficient obtained was only 0.69. A Hill coefficient significantly less than 1 is typical of binding events which exhibit negative cooperativity, where binding of substrate to one binding site makes binding of another substrate less likely. This result suggests some interplay between the protomers within the trimeric GltPh when it is reconstituted into a proteoliposome. In this case ligand binding could be associated with an actual transport process across the proteoliposome membrane leading to a difference in environment on either side of the membrane, as would be expected under physiological conditions.

Using the STD NMR method presented here, a constant protein concentration is maintained throughout the entire experiment. Determination of the apparent *K*_D_ does thus not rely on accurately knowing the protein concentration, which is often difficult to obtain for membrane proteins. The STD NMR approach therefore presents a simpler route for *K*_D_ estimation as compared with other methods commonly used^10,12,15–18^. Indeed, if we deliberately use a wrong estimation of the protein concentration (by a factor of two) to perform the calculations there is no difference in the *K*_D_ determined using this method. The two essential factors are to maintain a protein concentration below the expected range of the *K*_D_ of the reporter ligand (weak binder) and also to ensure that the protein concentration is kept constant. Thus, this is the first simple and quick analytical estimation of a ligand *K*_D_ to a membrane protein using STD NMR. Further, this method was expanded to include an accurate *K*_D_ estimation for a tightly binding ligand normally not accessible with STD-NMR, as well as the observation of important functional differences of a membrane protein reconstituted in different membrane mimetics, making this technique much more universally applicable.

This simplified approach is now being applied to other membrane proteins for both ligand and inhibitor studies. In less favourable cases where interferences from the strong detergent signals are present, perdeuterated detergent molecules could be used or double difference techniques (STDD NMR) applied^23^. One further alternative is to perform similar studies on membrane proteins encapsulated in novel membrane protein scaffolds (SMALPS) or indeed in whole cells^42^. Such experiments are currently being performed in our lab.

In summary, we have demonstrated a fast, STD NMR-based protocol for the determination of ligand binding constants across at least four orders of magnitude for membrane proteins. This experimental approach can be applied to both detergent-solubilised and proteoliposome-reconstituted proteins leading to important physiologically relevant information. Further, with improved spectrometer performance and sensitivity such competition STD NMR techniques could lead to important new applications such as lead discovery in which compound libraries are screened against a range of drug targets to identify both high- and low-affinity ligands. Analogues may then be rapidly rank-ordered, and structure–activity relationships derived that can be used to optimise such NMR “hits” into viable drug leads. Hence, the STD NMR approach described here could ultimately be considered as a powerful additional screening tool for future structure-based and fragment-based drug design (SBDD and FBDD respectively) especially in these and other key membrane protein systems.

## Materials and methods

### Plasmids

GltPh (wild type) inserted into pBAD24 plasmid was created by Eric Gouaux (Oregon Health and Science University, USA) and supplied by Harald H. Sitte and Thomas Stockner (Medical University of Vienna, Vienna, Austria)^18^.

### Expression and purification of GltPh in detergent

Plasmids including wild type (WT) GltPh were used to transform chemically competent TOP10 *Escherichia coli* bacterial cells (1 ng.μL^−1^, 100 mL). Cells were grown in LB medium up to an optical density of 0.7–0.8 at 600 nm (37 °C, 190 rpm). Protein production was induced by addition of 0.2% L-arabinose and cell harvesting was carried out after 3 h of incubation (30 °C, 190 rpm). Cells were pelleted by centrifugation (Thermo Scientific Sorvall RC6 Plus) at 6000 rcf for 8 min. The cells were re-suspended in lysis buffer (20 mM HEPES pH 7.5, 200 mM NaCl, 5 mM MgCl_2_) with DNAse, PMSF, lysozyme and glycerol, and lysis was performed using a high pressure EmulsiFlex system at 10,000 psi. Removal of all cellular debris was carried out via centrifugation (6000 rcf, 10 min). The resulting supernatant was then subjected to ultracentrifugation (176,000 rcf, 2 h) and the membrane pellet was re-suspended in lysis buffer. n-Dodecyl-ß-D-Maltoside Detergent (DDM) was added to a final concentration of 1%. The solution was left for 90 min at 4 °C with gentle agitation, after which the total detergent concentration was diluted to 0.5% and insoluble protein/protein aggregates were isolated via ultracentrifugation (176,000 rcf, 15 min).

For protein binding and purification, imidazole (pH 8.0) was added to a final concentration of 10 mM, and the solution was incubated with TALON Metal Affinity Resin overnight at 4 °C, under mild agitation. Washes 1 and 2 were carried out in 40 and 5 column volumes of buffer A (20 mM HEPES pH 7.5, 200 mM KCl, 0.075% DDM) supplemented with 10 mM and 20 mM imidazole respectively. Protein was eluted in 3 column volumes of buffer A supplemented with 500 mM imidazole. The purity of the eluted protein was analysed via SDS-PAGE gel electrophoresis. The eluted protein was exchanged to buffer B (20 mM HEPES pH 7.5, 100 mM KCl, 0.075% DDM) and afterwards concentrated using Amicon Ultra-15 Centrifugal Filter Units (100 kDa MWCO). The protein concentration was calculated by measuring the optical absorbance at 280 nm and using the molar extinction coefficient of WT GltPh. The purified protein was kept in 20% glycerol, aliquoted, flash frozen and stored at − 80 °C.

WT GltPh in buffer B with 20% glycerol was washed using a PD-10 desalting column prior to NMR experiments, to remove glycerol and other small molecule impurities. Buffers C (20 mM potassium phosphate pH 7.5, 100 mM NaCl, 0.075% DDM) and D (20 mM potassium phosphate pH 7.5, 100 mM KCl, 0.075% DDM) were prepared in D2O. Washed WT GltPh was prepared in buffer C via buffer exchange using an Amicon Ultra-4 Centrifugal Filter Unit (100 kDa MWCO), with a final protein concentration of 19.5 μM. Buffer exchange was repeated 4 times for maximum efficiency. Washed WT GltPh was additionally prepared in buffer D, to a concentration of 22.7 μM, ready for control NMR experiments in the absence of Na^+^ ions.

### Reconstitution of GltPh into liposomes

For reconstitution experiments WT GltPh was exchanged into buffer E (20 mM potassium phosphate buffer pH 7.5, 100 mM KCl, 0.075% DDM) and concentrated to approximately 50 μM. *Escherichia coli* polar lipid extract (Avanti Polar Lipids Inc.) and 1,2-dioleoyl-*sn*-glycero-phosphocholine (DOPC) (Avanti Polar Lipids Inc.) were mixed in a 1:3 ratio. The mixture was dried under a constant steady stream of argon to form a thin lipid film. The dried lipid mixture was then resuspended in degassed buffer F (20 mM potassium phosphate buffer pH 7.5, 100 mM KCl) to a final concentration of 20 mg.mL^−1^. The lipid suspension was subjected to 5 freeze–thaw cycles using liquid nitrogen, thawing at room temperature, resulting in the formation of large multilamellar vesicles (LMVs). The mixture was extruded through polycarbonate membranes with a pore size of 400 nm (Avanti Polar Lipids Inc.) to form large unilamellar vesicles (LUVs). The liposomes were diluted to a lipid concentration of 4 mg.mL^−1^ using buffer G (20 mM potassium phosphate buffer pH 7.5, 100 mM KCl, 25% glycerol). Liposomes were destabilised by dropwise addition of Triton X-100 (10% w/v), adding 150 μL in total per 5 mL of liposomes. WT GltPh in buffer E was added to the destabilised liposomal mixture at 20 μg protein per mg of lipid and left to agitate gently for 30 min. Detergent was removed by stepwise addition of Bio-Beads SM-2. The proteoliposome mixture was incubated and gently mixed with four successive additions of 200 mg Bio-Beads SM-2 per 5 mL of proteoliposome mixture, which were then removed by filtration. Liposomes were concentrated by centrifugation at 140,000 × *g* for 2 h at 4 °C. Buffers H (20 mM potassium phosphate pH 7.5, 100 mM NaCl) and I (20 mM potassium phosphate pH 7.5, 100 mM KCl) were prepared in D_2_O. Liposomes were resuspended in either buffer H or I to a final lipid concentration of approximately 40 mg.mL^−1^. Proteoliposomes (PL-WT GltPh) were used immediately for NMR experiments, and not flash frozen between times.

Empty liposomes were also prepared in the same manner and at the same lipid concentration, however steps involving liposome destabilising, protein addition and removal of detergent using Bio-Beads SM-2 were removed in this protocol. These served as control samples. The external buffer H was used for resuspension of the liposomes in the final step.

### NMR spectroscopy

The NMR spectra were recorded at 278 K with D_2_O as solvent, on an Ultra- Compact 800 MHz Bruker Avance III NMR spectrometer equipped with an inverse triple resonance (H/C/N) z-gradient probe head. STD NMR experiments were carried out by a pseudo 2D pulse sequence including spoil pulses to destroy residual magnetization during the relaxation delay (two trim pulses of 2.5 and 5 ms, followed by a 3 ms gradient pulse on the Z-axis). For selective saturation of GltPh, cascades of 49 ms Gaussian-shaped pulses (field strength of 90 Hz) were used with a 1 ms delay between successive pulses. Total saturation time for STD measurements was 2 s with a recycling delay of 2 s in experiments consisting of 512 scans. The saturation time of 2 s was selected as a compromise between sensitivity and accuracy on *K*_D_ determination, as a long full initial-slope STD NMR approach^38^ is not appropriate for relatively fast-degrading membrane proteins. Selective saturation of the protein was achieved by setting the on-resonance frequency at 0.6 ppm, to produce efficient saturation of the aliphatic side chains of the protein. This saturating radio frequency indeed also directly hit on the most up-field proton signal of the detergent molecules, contributing to a very efficient saturation of the whole system. Potential saturation transfer effects arising from non-specific interactions of the ligands with the detergent could be ruled out by negative results of STD NMR experiments of the protein–ligand system in the absence of Na^+^. For the reference (off-resonance) spectrum, the irradiation frequency was shifted to 40 ppm.

In the case of detergent solubilised WT GltPh, a series of STD NMR experiments were first carried out using WT protein in buffer C and increasing L-glutamate concentrations ranging from 0 to 5 mM (example NMR spectra in Figure S2). A control STD NMR measurement was then undertaken using detergent solubilised WT GltPh in buffer D (without Na^+^ ions) supplemented with 0.5 mM L-glutamate (Figure S1). Finally, a series of STD NMR experiments were taken following a competition-based titration of L-aspartate (ranging from 0 to 1 μM) into WT GltPh in buffer C supplemented with 1.5 mM L-glutamate.

For PL-WT GltPh, the first set of STD NMR experiments were carried out using proteoliposomes in external buffer H and increasing L-glutamate concentrations ranging from 0 to 5 mM (Figure S5). Two control STD NMR experiments were also undertaken, using (1) empty liposomes in external buffer H (Figure S3) and (2) PL-WT GltPh in external buffer I (Figure S4), each supplemented with 1.5 mM L-glutamate. Finally, a series of STD NMR experiments were taken following a competition-based titration of L-aspartate (ranging from 0 to 2 μM) into PL-WT GltPh in buffer H supplemented with 1.5 mM L-glutamate. Bruker Topspin (version 3.5) was employed for processing the NMR spectra and determination of peak integrals for STD-AF calculations as described in reference 21. All figures, except Fig. 1, were prepared using Matlab. The *K*_D_ determination was performed using the curve fitting tool in Matlab.

Spin–lattice relaxation times (*T*_1_) were determined using the inversion-recovery method^43^ for (a) 1.5 mM L-glutamate in buffer C, and (b) 1.5 mM L-glutamate added to empty liposomes in external buffer H. For these experiments, spectra of 16 K data points were obtained for 16 and 12 delay time (τ) values in cases (a) and (b) respectively, ranging from 1 ms to 5 s. Initial quick *T*_1_ estimation was performed for all samples in order to set the appropriate relaxation delay, which was set to 5 s in all samples. *T*_1_ values were obtained by fitting the data (Figure S6, table S1) to the following equation:

5 $$I={I}_{max}\left(1-2{e}^{{-\tau}/{T}_{1}}\right)$$

## Supplementary information

Supplementary file1

## Data Availability

The data sets generated during and/or analysed during the current study are available from the corresponding author on reasonable request.

## References

[1] Danbolt NC (2001). Glutamate uptake. Prog. Neurobiol..

[2] Owe SG, Marcaggi P, Attwell D (2006). The ionic stoichiometry of the GLAST glutamate transporter in salamander retinal glia. J. Physiol..

[3] Zerangue N, Kavanaugh MP (1996). Flux coupling in a neuronal glutamate transporter. Nature.

[4] Fairman WA, Vandenberg RJ, Arriza JL, Kavanaught MP, Amara SG (1995). An excitatory amino-acid transporter with properties of a ligand-gated chloride channel. Nature.

[5] Canul-Tec JC (2017). Structure and allosteric inhibition of excitatory amino acid transporter 1. Nature.

[6] Yernool D, Boudker O, Jin Y, Gouaux E (2004). Structure of a glutamate transporter homologue from *Pyrococcus horikoshii*. Nature.

[7] Jensen S, Guskov A, Rempel S, Hänelt I, Slotboom DJ (2013). Crystal structure of a substrate-free aspartate transporter. Nat. Struct. Mol. Biol..

[8] Garaeva AA (2018). Cryo-EM structure of the human neutral amino acid transporter ASCT2. Nat. Struct. Mol. Biol..

[9] Ji Y, Postis VLG, Wang Y, Bartlam M, Goldman A (2016). Transport mechanism of a glutamate transporter homologue GltPh. Biochem. Soc. Trans..

[10] Boudker O, Ryan RM, Yernool D, Shimamoto K, Gouaux E (2007). Coupling substrate and ion binding to extracellular gate of a sodium-dependent aspartate transporter. Nature.

[11] Reyes N, Ginter C, Boudker O (2009). Transport mechanism of a bacterial homologue of glutamate transporters. Nature.

[12] Akyuz N (2015). Transport domain unlocking sets the uptake rate of an aspartate transporter. Nature.

[13] Verdon G, Boudker O (2012). Crystal structure of an asymmetric trimer of a bacterial glutamate transporter homolog. Nat. Struct. Mol. Biol..

[14] Psakis G (2007). Expression screening of integral membrane proteins from *Helicobacter pylori* 26695. Protein Sci..

[15] Hänelt I, Jensen S, Wunnicke D, Slotboom DJ (2015). Low affinity and slow Na+ binding precedes high affinity aspartate binding in the secondary-active transporter GltPh. J. Biol. Chem..

[16] Silverstein N, Ewers D, Forrest LR, Fahlke C, Kanner BI (2015). Molecular determinants of substrate specificity in sodium-coupled glutamate transporters. J. Biol. Chem..

[17] Reyes N, Oh S, Boudker O (2013). Binding thermodynamics of a glutamate transporter homolog. Nat. Struct. Mol. Biol..

[18] Venkatesan SK (2015). Refinement of the central steps of substrate transport by the aspartate transporter GltPh: Elucidating the role of the Na2 sodium binding site. PLoS Comput. Biol..

[19] Mayer M, Meyer B (1999). Characterization of ligand binding by saturation transfer difference NMR spectroscopy. Angew. Chemie - Int. Ed..

[20] Mayer M, Meyer B (2001). Group epitope mapping by saturation transfer difference NMR to identify segments of a ligand in direct contact with a protein receptor. J. Am. Chem. Soc..

[21] Viegas A, Manso J, Nobrega FL, Cabrita EJ (2011). Saturation-transfer difference (STD) NMR: A simple and fast method for ligand screening and characterization of protein binding. J. Chem. Educ..

[22] Mari S, Serrano-Gómez D, Cañada FJ, Corbí AL, Jiménez-Barbero J (2004). 1D saturation transfer difference NMR experiments on living cells: The DC-SIGN/oligomannose interaction. Angew. Chemie Int. Ed..

[23] Claasen B, Axmann M, Meinecke R, Meyer B (2005). Direct observation of ligand binding to membrane proteins in living cells by a saturation transfer double difference (STDD) NMR spectroscopy method shows a significantly higher affinity of integrin αIIbβ 3 in native platelets than in liposomes. J. Am. Chem. Soc..

[24] Airoldi C, Giovannardi S, Laferla B, Jiménez-Barbero J, Nicotra F (2011). Saturation transfer difference NMR experiments of membrane proteins in living cells under HR-MAS conditions: The interaction of the SGLT1 co-transporter with its ligands. Chem. A Eur. J..

[25] Cox BD (2015). Structural analysis of CXCR4—Antagonist interactions using saturation-transfer double-difference NMR. Biochem. Biophys. Res. Commun..

[26] Venkitakrishnan RP, Benard O, Max M, Markley JL, Assadi-Porter FM (2012). Use of NMR Saturation Transfer Difference Spectroscopy to Study Ligand Binding to Membrane Proteins. Membrane Protein Structure and Dynamics.

[27] Meinecke R, Meyer B (2001). Determination of the binding specificity of an integral membrane protein by saturation transfer difference NMR: RGD peptide ligands binding to integrin αIIbβ3. J. Med. Chem..

[28] Fredriksson K (2017). Nanodiscs for INPHARMA NMR characterization of GPCRs: Ligand binding to the Human A2A Adenosine Receptor. Angew. Chemie Int. Ed..

[29] Igonet S (2018). Enabling STD-NMR fragment screening using stabilized native GPCR: A case study of adenosine receptor. Sci. Rep..

[30] Yong KJ (2018). Determinants of ligand subtype-selectivity at α 1A-Adrenoceptor revealed using saturation transfer difference (STD) NMR. ACS Chem. Biol..

[31] Vaid TM, Chalmers DK, Scott DJ, Gooley P (2020). INPHARMA based determination of ligand binding modes at α1-adrenergic receptors explains the molecular basis of subtype selectivity. Chem. A Eur. J..

[32] Bumbak F (2020). Conformational changes in tyrosine 11 of neurotensin are required to activate the neurotensin receptor 1. ACS Pharmacol. Transl. Sci..

[33] Koch HP, Larsson HP (2005). Small-scale molecular motions accomplish glutamate uptake in human glutamate transporters. J. Neurosci..

[34] Grewer C (2005). The individual subunits of the glutamate transporter EAAC1 homotrimer function independently of each other. Biochemistry.

[35] Erkens GB, Hänelt I, Goudsmits JMH, Slotboom DJ, Van Oijen AM (2013). Unsynchronised subunit motion in single trimeric sodium-coupled aspartate transporters. Nature.

[36] Ruan Y (2017). Direct visualization of glutamate transporter elevator mechanism by high-speed AFM. Proc. Natl. Acad. Sci. USA..

[37] Claridge TDW (2016). High-Resolution NMR Techniques in Organic Chemistry. Protein-Ligand Screening by NMR.

[38] Angulo J, Enríquez-Navas PM, Nieto PM (2010). Ligand-receptor binding affinities from saturation transfer difference (STD) NMR. Chem. A Eur. J..

[39] Wang Y, Sen Liu D, Wyss DF (2004). Competition STD NMR for the detection of high-affinity ligands and NMR-based screening. Magn. Reson. Chem..

[40] Cantor CR, Schimmel PR (1980). Biophysical Chemistry: Part III. Ligand interactions at equilibrium.

[41] Cheng Y, Prusoff WH (1973). Relationship between the inhibition constant (KI) and the concentration of inhibitor which causes 50 per cent inhibition (I50) of an enzymatic reaction. Biochem. Pharmacol..

[42] Postis V (2015). The use of SMALPs as a novel membrane protein scaffold for structure study by negative stain electron microscopy. Biochim. Biophys. Acta Biomembr..

[43] Vold RL, Waugh JS, Klein MP, Phelps DE (1968). Measurement of spin relaxation in complex systems. J. Chem. Phys..

