# Supplementary material for: Saturation transfer difference NMR on the integral trimeric membrane transport protein GltPh determines cooperative substrate binding
Source: Sci Rep. 2020 Oct 5;10:16483. doi: 10.1038/s41598-020-73443-z (PMC7536232; doi:10.1038/s41598-020-73443-z)

Supplementary Information

**Saturation transfer difference NMR on the integral trimeric membrane transport protein GltPh determines cooperative substrate binding**

Jenny L. Hall^a^, Azmat Sohail^b^, Eurico J. Cabrita^c^, Colin Macdonald^a^, Thomas Stockner^b^, Harald H. Sitte^b^, Jesus Angulo^d^, & Fraser MacMillan*^,a^

^a^Henry Wellcome Unit for Biological EPR, School of Chemistry, Norwich Research Park University of East Anglia, Norwich NR4 7TJ, United Kingdom,

^b^ Institute of Pharmacology, Medical University of Vienna, Währingerstrasse 13A, A-1090 Vienna, Austria,

^c^ UCIBIO, Chemistry Department, Faculty of Sciences and Technology, NOVA University of Lisbon, 2829-516 Caparica, Portugal,

^d^ School of Pharmacy, University of East Anglia, Norwich Research Park, Norwich, NR4 7TJ, UK.

**Abstract**

Saturation-transfer difference (STD) NMR spectroscopy is a fast and versatile method which can be applied for drug-screening purposes, allowing the determination of essential ligand binding affinities (K_D_). Although widely employed to study soluble proteins, its use remains negligible for membrane proteins. Here the use of STD NMR for K_D_ determination is demonstrated for two competing substrates with very different binding affinities (low nanomolar to millimolar) for an integral membrane transport protein in both detergent-solubilised micelles and reconstituted proteoliposomes. GltPh, a homotrimeric aspartate transporter from *Pyrococcus horikoshii*, is an archaeal homolog of mammalian membrane transport proteins – known as excitatory amino acid transporters (EAATs). They are found within the central nervous system and are responsible for fast uptake of the neurotransmitter glutamate, essential for neuronal function. Differences in both K_D_’s and cooperativity are observed between detergent micelles and proteoliposomes, the physiological implications of which are discussed.

Supplementary Figures

**Figure S1.** Full reference ^1^H NMR (top) and corresponding STD NMR spectra (bottom) of 22.7 μM detergent solubilised GltPh incubated with 0.5 mM L-glutamate in the absence of Na^+^ ions (800 MHz, 5 ^o^C, 2s saturation time). For the sake of comparison, the intensities are matched at the signal at 1.4 ppm. No signals from L-glutamate (neither from the small osmolytes present in the protein sample) are seen in the difference spectrum (bottom), indicating that no binding is occurring in solution. The strong signals in the STD spectrum correspond to the detergent n-dodecyl-β-D-maltoside.

**
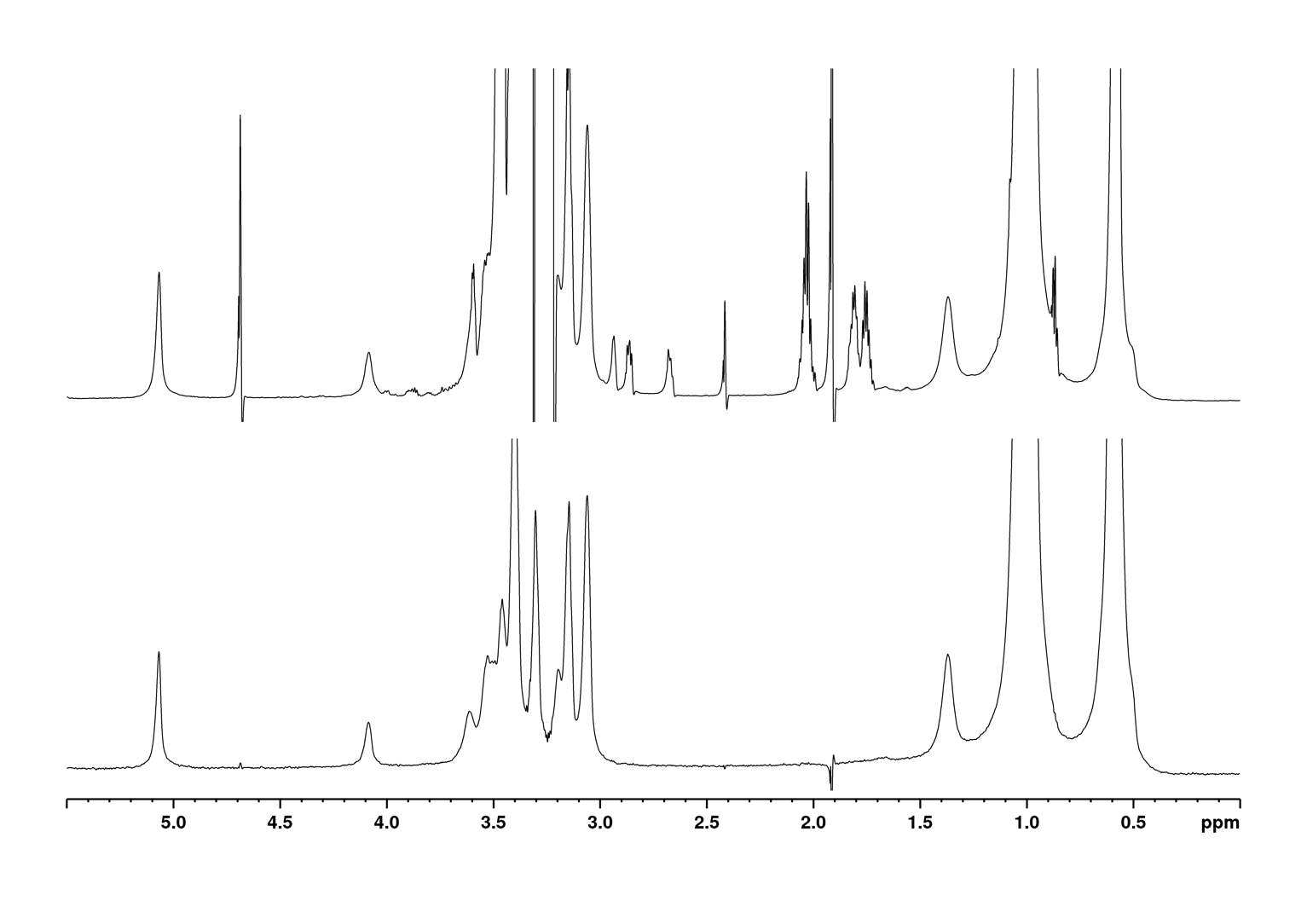
**

**
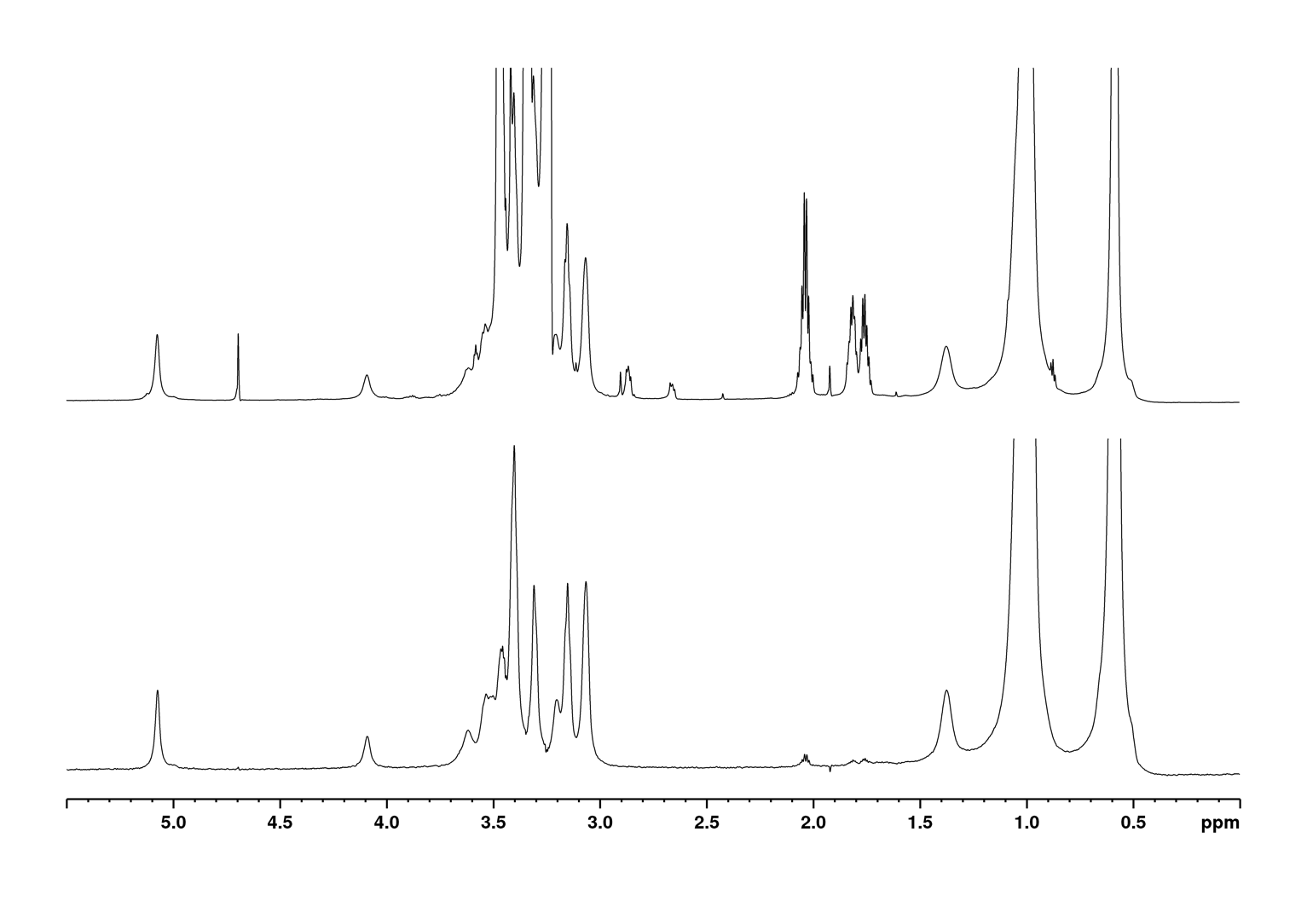
Figure S2.** Full reference ^1^H NMR (top) and corresponding STD NMR spectra (bottom) of 19.4 μM detergent solubilised GltPh incubated with 1.5 mM L-glutamate in the presence of Na^+^ ions (800 MHz, 5 ^o^C, 2s saturation time). For the sake of comparison, the intensities are matched at the signal at 1.4 ppm. Signals from L-glutamate are observed at 2.05 ppm and 1.8 ppm in the difference spectrum (bottom) reporting on the binding of the specific ligand to GltPh. No signals from osmolytes (region between 2.7 and 3.0 ppm) are seen in the STD spectrum.

**Figure S3.** Full reference ^1^H NMR (top) and corresponding STD NMR spectra (bottom) of empty liposomes incubated with 1.5 mM L-glutamate in the presence of Na^+^ ions (800 MHz, 5 ^o^C, 2s saturation time). No signals from L-glutamate are seen in the difference spectrum (bottom), indicating that non-specific interactions are not occurring between L-glutamate and the liposomes.

**
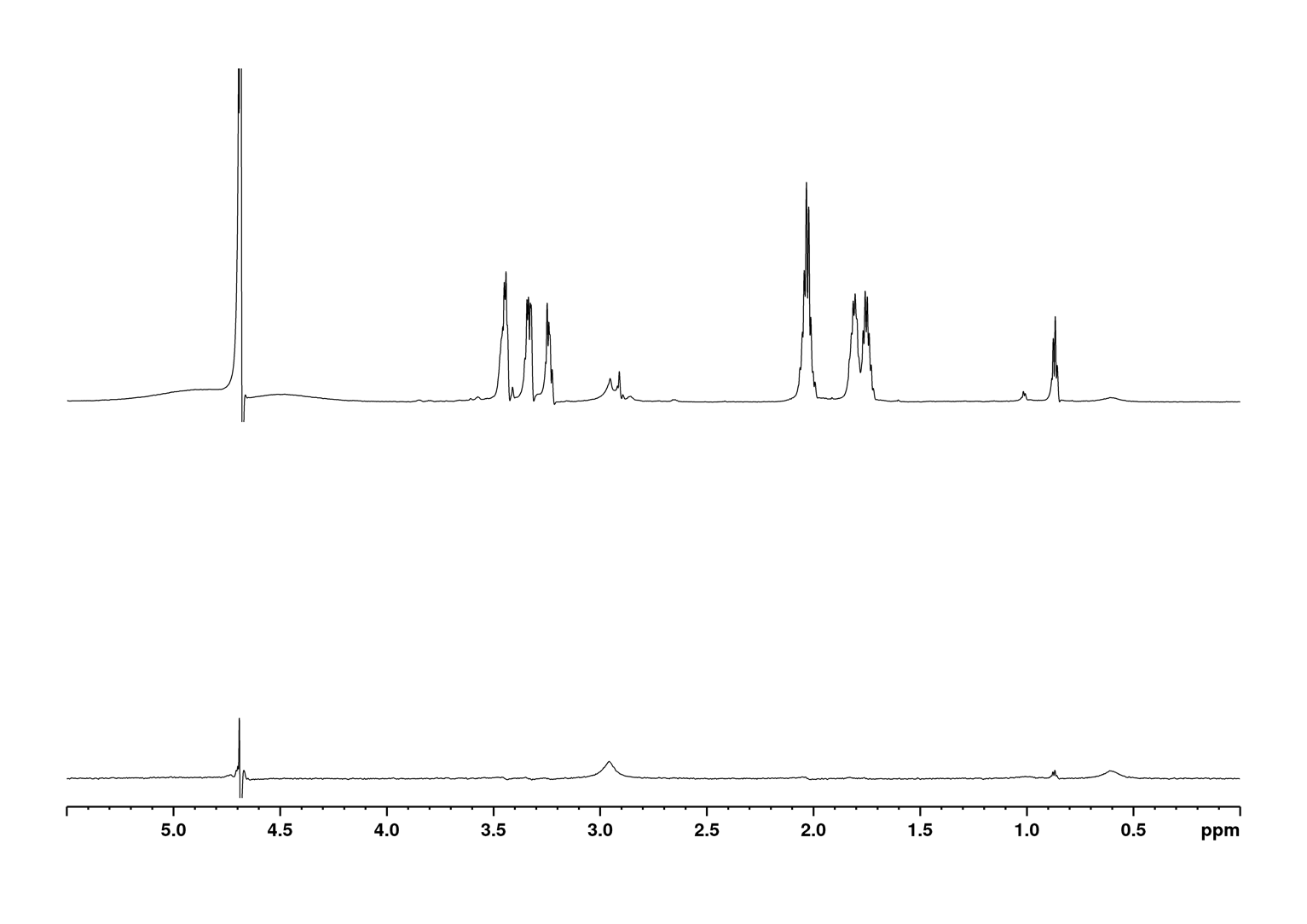
**

**
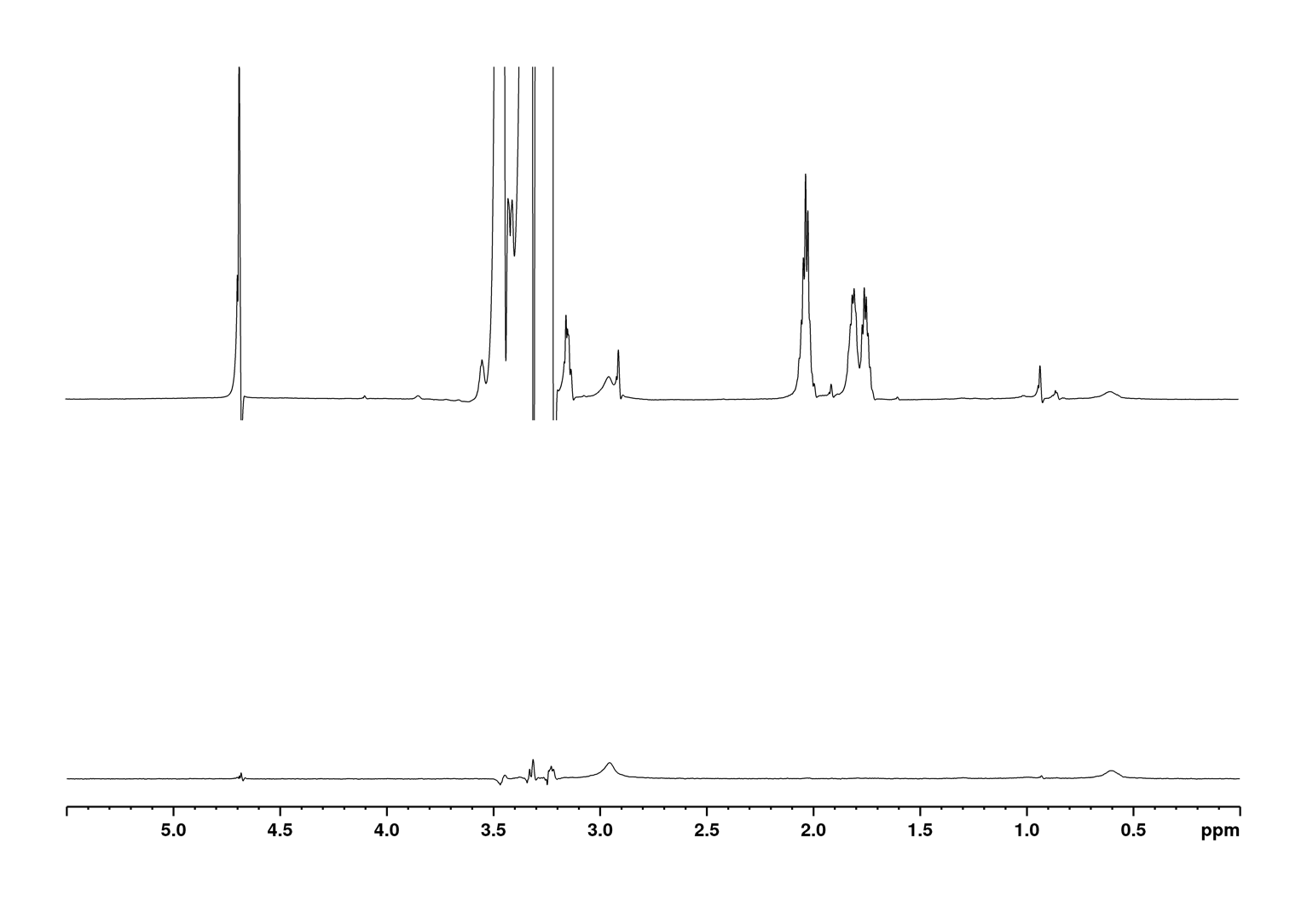
Figure S4.** Full reference ^1^H NMR (top) and corresponding STD NMR spectra (bottom) of WT GltPh reconstituted into liposomes, incubated with 1.5 mM L-glutamate in the absence of Na^+^ ions (800 MHz, 5 ^o^C, 2s saturation time). No signals from L-glutamate are seen in the difference spectrum (bottom), indicating that no binding is occurring in solution.

**
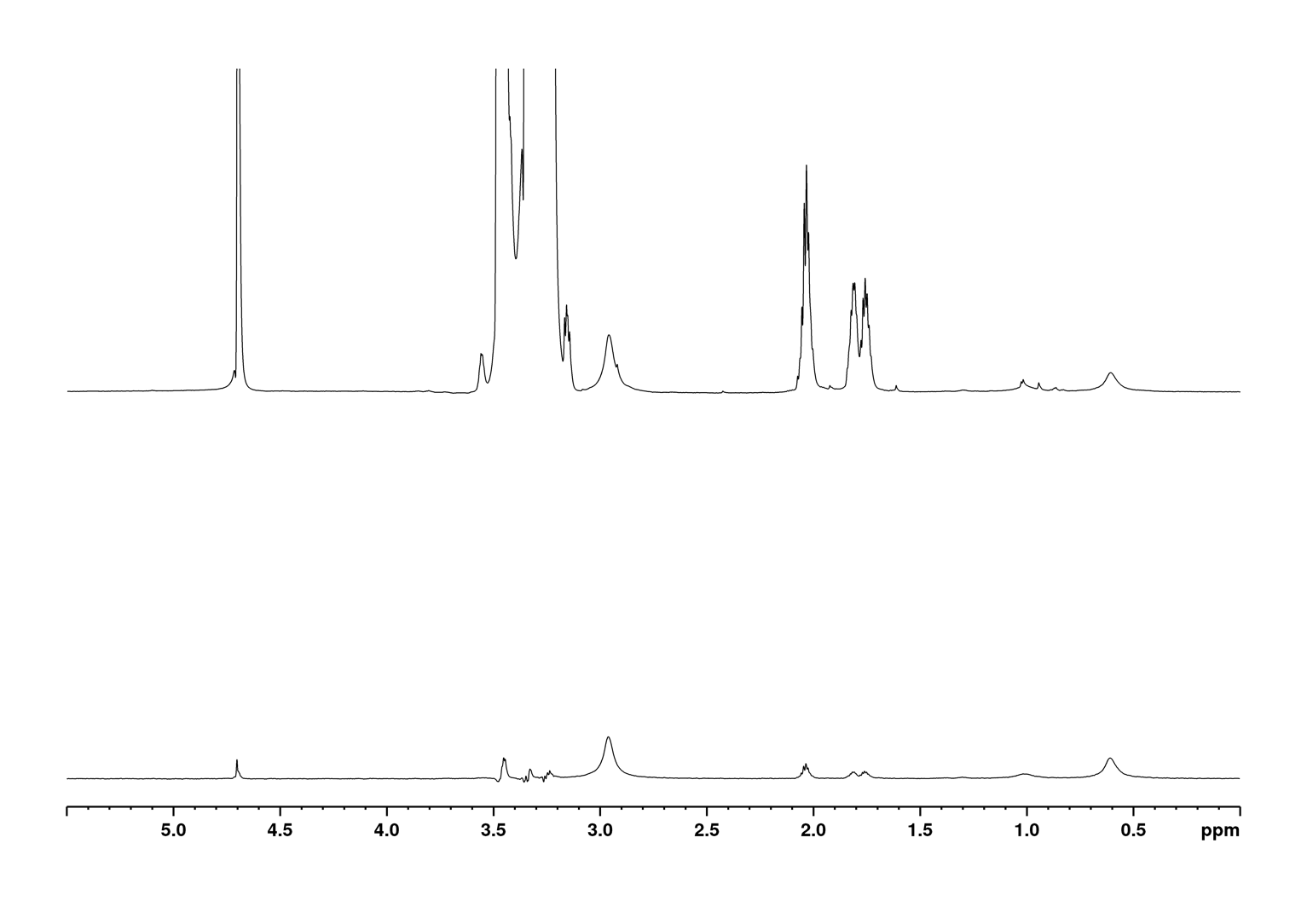
Figure S5.** Full reference ^1^H NMR (top) and corresponding STD NMR spectra (bottom) of WT GltPh reconstituted into liposomes, incubated with 1.5 mM L-glutamate in the presence of Na^+^ ions (800 MHz, 5 ^o^C, 2s saturation time). Signals from L-glutamate occur at 2.05 ppm and 1.8 ppm in the difference spectrum (bottom) reporting on the specific binding of the ligand to GltPh.


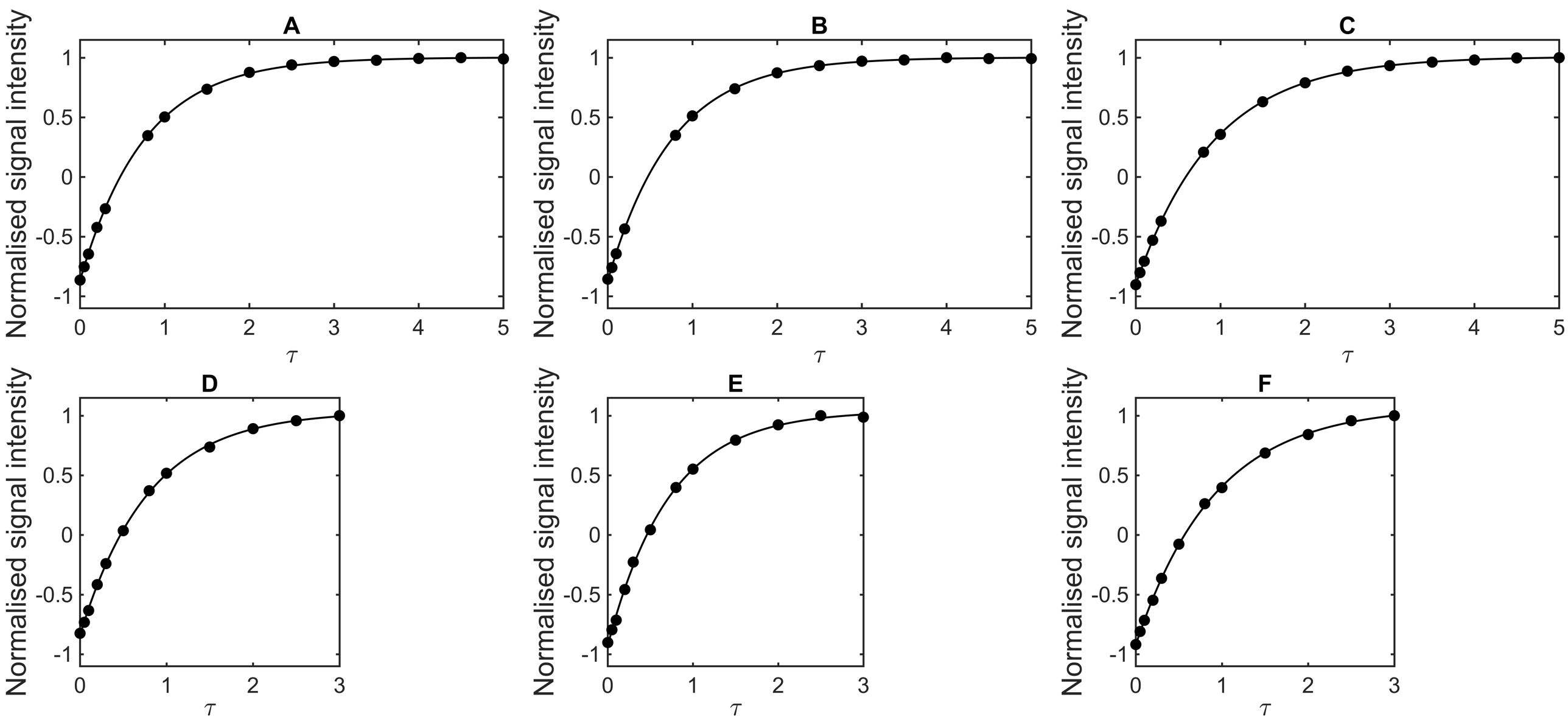
**Figure S6**. Plots of normalised ^1^H NMR signal intensity against delay time τ values (dots) and the associated fittings according to equation S1 (lines) for (top panel) 1.5 mM L-glutamate in buffer C and (bottom panel) 1.5 mM L-glutamate added to empty liposomes in external buffer H. Normalised signal intensities of L-glutamate protons at the γ position at 2.05 ppm (A and D), the β position at 1.81 ppm (B and E) and the second β position at 1.75 ppm (C and F) are plotted.

**Table S1.** Table of the determined T_1_ values for each observable L-glutamate proton resonance calculated according to equation S1.

|  | T_1_ (ms) | | |
| --- | --- | --- | --- |
|  | γ proton (2.05 ppm) | β proton (1.81 ppm) | β proton (1.75 ppm) |
| 1.5 mM L-glu in buffer C | 923.4 | 755.0 | 758.8 |
| 1.5 mM L-glu + empty liposomes in external buffer H | 914.2 | 718.3 | 786.3 |

$I=I_{max}\left( 1-2e^{\frac{-\tau}{T_{1}}} \right)$ (S1)

**Figure S7**. Plot of the STD effect (%) against the Na^+^ ion concentration in 14 μM detergent-solubilised GltPh incubated with 1.5 mM L-glutamate.


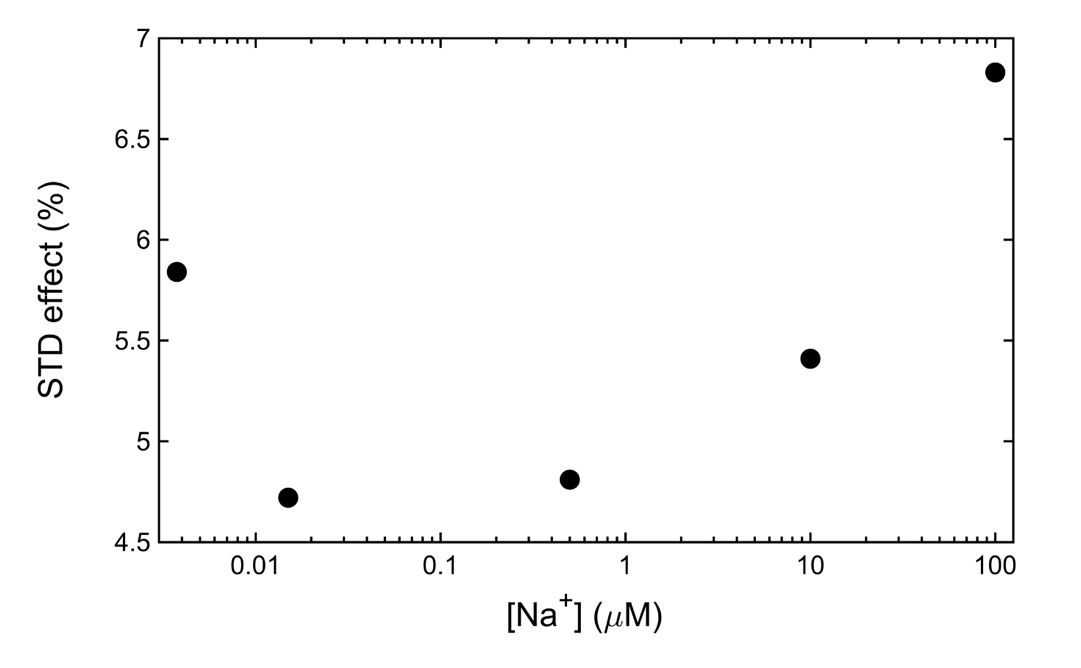

Supplement: Supplementary file 1 — Supplementary file1 [file 41598_2020_73443_MOESM1_ESM.docx]
